# Expression of concern for Euro Surveill. 2026;31(28)

**DOI:** 10.2807/1560-7917.ES.2026.31.36.260910ec

**Published:** 2026-09-10

**Authors:** Eurosurveillance editorial team

**Affiliations:** 1 European Centre for Disease Prevention and Control (ECDC), Stockholm, Sweden

Regarding the article "Trends in syphilis, gonorrhoea and chlamydia: a descriptive analysis of national surveillance data, Poland, 2013 to 2024" by Kadylak et al, published on 16 July 2026, the corresponding author has informed *Eurosurveillance* of data handling errors which affected the presented incidences and per cent changes for all three diseases. We are currently investigating the situation related to this honest error and will take further steps as necessary. 

While the investigation is ongoing, the editors decided to publish an expression of concern for this article. We are grateful to the authors for flagging the issue in a timely manner.

